# Clinical characteristics and treatment outcomes of cervical high-grade squamous intraepithelial lesions in women under 25 years: a retrospective cohort study

**DOI:** 10.3389/fonc.2025.1700356

**Published:** 2025-11-26

**Authors:** Yanmei Liu, Ke Peng, Shujun Gao

**Affiliations:** 1 Department of Gynecology, Obstetrics and Gynecology Hospital of Fudan University, Shanghai, China; 2 Shanghai Key Laboratory of Female Reproductive Endocrine Related Diseases, Shanghai, China; 3 Center of Diagnosis and Treatment for Cervical & Uterine Cavity Disease, Obstetrics and Gynecology Hospital of Fudan University, Shanghai, China; 4 Department of Gynecology, The International Peace Maternity and Child Health Hospital, School of Medicine, Shanghai Jiao Tong University, Shanghai, China; 5 Shanghai Key Laboratory of Embryo Original Diseases, Shanghai, China

**Keywords:** cervical HSIL under 25 years old, clinical characteristics, treatment outcomes, CO2 laser, LEEP

## Abstract

**Objectives:**

Limited data are available regarding the clinical characteristics and treatment outcomes of cervical HSIL in women under 25, particularly in Asian populations. This study aimed to investigate the clinical features of cervical HSIL in women under 25 and to compare the efficacy of various treatment modalities, with the goal of informing early detection and optimal management strategies.

**Methods:**

A retrospective cohort study was conducted involving 210 patients under 25 who were diagnosed with cervical HSIL at Obstetrics and Gynecology Hospital of Fudan University between January 2019 and December 2021. Data on clinical features, cytology, HPV status, colposcopy findings, and treatment approaches were collected and analyzed. Treatment outcomes were assessed by comparing postoperative cytology, HPV clearance, and pathological results at 6 and 12 months among patients managed with observation, CO2 laser therapy, or LEEP.

**Results:**

Most patients (89.0%) were diagnosed via routine screening and 86.7% were asymptomatic. Among symptomatic cases, postcoital bleeding (10.0%) and abnormal vaginal discharge (3.3%) were reported. Cytology showed NILM in 38.1%, ASCUS 30.5%, LSIL 21.0%, HSIL 7.1%, and ASC-H 3.3%. HPV was detected in 98.1% of patients, predominantly HPV16 (65.2%). Cytology alone showed low sensitivity for detecting cervical HSIL (7.1%) compared with HPV testing (98.1%) or co-testing (98.6%, χ²=534.468, P<0.001). At 12-month follow-up, the LEEP group exhibited the highest rates of normal cytology (90.5%), HPV clearance (87.5%), and histologic cure (95.2%), outperforming both the laser and observation groups. Multivariate logistic regression analyses identified reproductive tract inflammation as an independent risk factor for persistent high-risk HPV infection among patients undergoing observation.

**Conclusions:**

Most cervical HSIL under 25 are asymptomatic. Cytology alone is insufficient for reliable detection, HPV based screening significantly improves sensitivity. Among treatment strategies, LEEP offers the most effective histological cure rate, although potential reproductive risks warrant careful consideration in nulliparous women.

## Introduction

Cervical cancer ranks as the fourth most common malignancy in women globally, imposing a substantial disease burden (1). Cervical squamous epithelial lesion (SIL), a recognized precursor to invasive carcinoma, is categorized by the 2014 WHO classification into low-grade squamous intraepithelial lesion (LSIL) and high-grade squamous intraepithelial lesion (HSIL) (2). Whereas LSIL often regresses spontaneously, persistent HSIL carries a significant risk of progression to invasive cancer. HSIL, encompassing cervical intraepithelial neoplasia (CIN) grade 2 and 3(CIN2, CIN3), is therefore regarded as a definitive precancerous lesion. Consequently, the early detection and management of HSIL are pivotal for reducing the incidence and mortality of cervical cancer.

Cervical cancer screening strategies have undergone significant evolution over recent decades. In 2012, the American Society for Colposcopy and Cervical Pathology (ASCCP) recommended initiating cytology screening from age 21, with co-testing (combined cytology and HPV testing) from age 30 (3). However, the sensitivity of cytology alone is suboptimal, and more than half of women aged 25–29 with histologically confirmed CIN3 or more severe lesions exhibit normal cytology results (4). In response, the ASCCP updated its guidelines in 2015, endorsing primary HPV testing as the preferred screening strategy starting at age 25 (5). Notably, high-risk HPV testing is not recommended for individuals under 25 years of age. This screening gap may lead to delayed diagnosis of HSIL and cervical cancer in younger women, compounding psychological and economic burdens. Although routine HPV testing is not indicated in this age group, a subset of symptomatic women under 25 are diagnosed with HSIL via colposcopy, often accompanied by considerable psychological distress.

Currently, systematic evidence regarding the clinical characteristics and management outcomes of HSIL in women younger than 25 remains limited. To address this knowledge gap, we conducted a retrospective study of HSIL in this population, analyzing data on cytology, HPV status, and colposcopic pathology across different management approaches—including observation, loop electrosurgical excision procedure (LEEP), and CO_2_ laser therapy. This study aims to provide evidence that informs early diagnosis and optimizes treatment strategies for this young patient population.

## Materials and methods

### Study design and participants

The retrospective study was conducted to investigate the clinical characteristics of cervical high-grade squamous intraepithelial lesion (HSIL) in women younger than 25 years and to compare the efficacy of different treatment. A total of 210 patients with cervical HSIL under 25 years were included.

Inclusion criteria were as follows: patients who underwent colposcopy at the Obstetrics and Gynecology Hospital of Fudan University between January 2019 and December 2021; age under 25 years; the cervical transformation zone was classified as Type I or Type II; pathological diagnosis of cervical HSIL via colposcopy; endocervical curettage (ECC) showed no pathological abnormality.

Exclusion criteria were as follows: patients with cervical HSIL aged ≥25 years; history of cervical epithelial lesions or cervical surgery; patients with conditions affecting communication, such as dementia, aphasia, deafness, or impaired consciousness; pregnant women; patients with severe medical or surgical diseases, major organ dysfunction, or other malignancies not in complete remission.

### Data collection

Clinical data of enrolled cases were collected, including age, reason for visit, clinical manifestations, cytology results, HPV results, distribution characteristics of the lesion and colposcopy pathological results. Efficacy assessment was performed at 6 and 12 months post-treatment (observation, CO2 laser, or LEEP) by comparing follow-up cytology, HPV testing, and colposcopic pathological outcomes across the three management approaches.

### CO2 laser ablation treatment

Laser vaporization of cervical HSIL lesions was performed using a CO_2_ laser device (Shanghai Laser Technology Research Institute Co., Ltd.) in continuous output mode. The procedure was conducted at a power setting of 25 W to achieve a vaporization depth of approximately 7–10 mm.

### LEEP procedure

The procedure was guided by preoperative colposcopic assessment of lesion extent, transformation zone type, and cervical size. The entire transformation zone was excised using a LiBang-200 LEEP system coupled with an SRD3000B high-frequency electrosurgical unit (Guangzhou San Rui Medical Equipment Co., Ltd.), with power settings of 60 W for cutting and 40 W for coagulation. Hemostasis was achieved via electrocoagulation, and the specimen was submitted for pathological examination.

### Follow-up

At the 6- and 12-month follow-ups subsequent to management (observation, CO_2_ laser, or LEEP), patients underwent a comprehensive follow-up comprising cervical cytology, HPV testing, and colposcopy with guided biopsy.

### Statistical analysis

Analysis was performed using SPSS (IBM SPSS Statistics for Windows, Version 25.0. IBM Corp. 2017. Armonk, New York, NY). Count data were expressed as number of cases (n) and percentage (%), and intergroup comparisons were performed using the chi-square (χ²) test, with a p-value < 0.05 considered statistically significant.

## Results

### Clinical characteristics

A total of 210 patients under 25 years of age with cervical HSIL were included in this study. The cohort comprised 23 (11.0%) patients aged 18–20 years and 187 (89.0%) aged 21–24 years. The primary reason for hospital presentation was routine gynecological examination, accounting for 90.0% of cases, while 10.0% of patients sought care due to postcoital bleeding. Among the participants, 182 (86.7%) were asymptomatic, 21 (10.0%) presented with postcoital bleeding, and 7 (3.3%) reported abnormal vaginal discharge. All patients with abnormal discharge were co-diagnosed with vaginitis.

Regarding obstetric history, 150 patients (71.4%) had no history of pregnancy, 37 (17.6%) had one prior pregnancy, and 23 (11.0%) had two or more pregnancies. In terms of delivery history, 192 patients (91.4%) were nulliparous, 14 (6.7%) had one prior delivery, and 4 (1.9%) had two or more deliveries.

Cytological evaluation indicated that NILM was the most common finding (38.1%), followed by ASCUS (30.5%), LSIL (21.0%), HSIL (7.1%), and ASC-H (3.3%). HPV testing was positive in 206 patients, yielding a high positivity rate of 98.1%, while only four patients tested negative. HPV16 was the most prevalent genotype (65.2%), followed by HPV18 (11.0%) and other 12 high-risk HPV types (49.5%). The HPV genotypes of 11 patients were unknown. Single-type HPV infection accounted for 50.5% of cases, while mixed infections comprised 41.9% (**Table 1**).

**Table 1:** Clinical characteristics of cervical HSIL patients under 25 years old.

Characteristics	No. patients (%)
Age (years) 18-20 21-24 Reason for visit Physical examination Postcoital bleeding	23 (11.0) 187 (89.0) 189 (90.0) 21 (10.0)
Clinical manifestations No Postcoital bleeding Abnormal vaginal discharge Gravidity (times) 0 1 2 3 4 6 Parity (times)	182 (86.7) 21 (10.0) 7 (3.3) 150 (71.4) 37 (17.6) 10 (4.8) 11 (5.2) 1 (0.5) 1 (0.5)
0 1 2 Cytology NILM ASCUS ASCU-H LSIL HSIL HPV Negative Positive HPV genotyping HPV16 HPV18 Other 12 types Unknown genotype HPV Infection classification Single infection Multiple infection Lesion distribution Focal Multifocal Lesion covering (quadrant) 1 ≥2	192 (91.4) 14 (6.7) 4 (1.9) 80 (38.1) 64 (30.5) 7 (3.3) 44 (21.0) 15 (7.1) 4 (1.9) 206 (98.1) 137 (65.2) 23 (11.0) 104 (49.5) 11 (5.2) 106 (50.5) 88 (41.9) 10 (4.8) 200 (95.2) 22 (10.5) 188 (89.5)

NILM, Negative for intraepithelial lesion or malignancy; ASC-US, atypical Squamous Cells of Undetermined Significance; ASCU-H, cannot exclude HSIL atypical squamous cells; LSIL, low-grade squamous intraepithelial lesion; HSIL, high-grade squamous intraepithelial lesion; other 12 types of HPV genotype include HPV31,33,35,39,45,51,52,56,58,59,66 and 68.

HSIL lesion distribution was predominantly multifocal (95.2%), with only 4.8% being focal. Furthermore, the majority of patients (89.5%) had lesions involving two or more cervical quadrants (**Table 1**).

### Sensitivity of screening methods for detecting cervical HSIL

Cervical cytology demonstrated a sensitivity of only 7.1% for detecting cervical HSIL, in contrast to 98.1% for high-risk HPV testing—a difference that was statistically significant (χ² = 348.394, P < 0.001). Co-testing with HPV and cytology reached a sensitivity of 98.6%, which was also significantly higher than that of cytology alone (χ² = 352.236, P < 0.001). However, no statistically significant difference in sensitivity was observed between HPV testing alone and co-testing (χ² = 0.145, P = 0.703) (**Table 2**).

**Table 2:** Sensitivity of various screening methods for detecting cervical HSIL.

Screening methods	Sensitivity	P-value	χ²
TCT	7.1%		
HPV	98.1%	< 0.001	534.468
TCT combined HPV	98.6%		

### Outcomes at 6 and 12 months post-treatment with different therapeutic regimens

Among 210 patients under 25 years of age with cervical HSIL, colposcopic biopsy confirmed HSIL (unspecified grade) in 168 cases and CIN2 in 42 cases. Following comprehensive counseling on the risks and benefits of available management options—including loop electrosurgical excision procedure (LEEP), CO_2_ laser ablation, and observation—all 168 patients with unspecified HSIL elected to undergo LEEP. Of the 42 patients with CIN2, 27 chose CO_2_ laser ablation and 15 opted for observation.

In the LEEP group, postoperative histology confirmed HSIL in 149 cases (88.7%), LSIL in 14 (8.3%), and normal cervical tissue in 5 (3.0%). Positive surgical margins were identified in 15 patients (8.9%), all located on the ectocervix, the remaining 153 (91.1%) had negative margins.

At the 6-month follow-up, normal cytology rates were 87.5% in the LEEP group, compared with 70.4% in the laser group (χ² = 5.319, P = 0.036). The observation group had a significantly lower normal cytology rate of 53.3% (χ² = 14.718, P = 0.002 *vs*. both active treatments). HPV clearance rates were 64.9% in the LEEP group and 55.6% in the laser group, both significantly higher than the 13.3% observed in the observation group (χ² = 15.501, P < 0.001), with no significant difference between LEEP and laser groups (χ² = 0.874, P = 0.392). The histological cure rate was highest in the LEEP group (94.0%), significantly exceeding that of the laser (77.8%) and observation (53.3%) group (χ² = 27.440, P < 0.001). No significant difference was observed between the laser and observation groups (χ² = 2.696, P = 0.163). Among the 15 patients in under observation, 6-month biopsy results showed normal cervical tissue in 8 (53.3%), LSIL in 1 (6.7%), and persistent CIN2 in 6 (40.0%) (**Table 3**).

**Table 3:** Cytological results, HPV status, and pathological results in cervical HSIL patients under 25 years old following LEEP, laser therapy, or observation.

Follow-up time and content	LEEP n (%)	CO2 laser n (%)	Observation n (%)	P-value	χ²
At 6 Months					
Cytology					
NILM	147 (87.5)	19 (70.4)	8 (53.3)	0.002	14.718
ASCUS/LSIL	21 (12.5)	8 (29.6)	7 (46.7)		
HPV					
Negative	109 (64.9)	15 (55.6)	2 (13.3)	< 0.001	15.501
Positive	59 (35.1)	12 (44.4)	13 (86.7)		
Colposcopy pathology					
Normal	158 (94.0)	21 (77.8)	8 (53.3)	< 0.001	27.440
LSIL/HSIL	10 (6.0)	6 (22.2)	7 (46.7)		
At 12 Months					
Cytology					
NILM	152 (90.5)	22 (81.5)	10 (66.7)	0.018	8.272
ASCUS/LSIL	16 (9.5)	5 (18.5)	5 (33.3)		
HPV					
Negative	147 (87.5)	19 (70.4)	9 (60.0)	0.005	11.247
Positive	21 (12.5)	8 (29.6)	6 (40.0)		
Colposcopy pathology					
Normal	160 (95.2)	24 (88.9)	10 (66.7)	0.001	16.508
LSIL/HSIL	8(4.8)	3 (11.1)	5 (33.3)		

At 12 months, normal cytology rates were 90.5% in the LEEP group, 81.5% in the laser group and 66.7% in the observation group (χ² = 8.272, P = 0.018). HPV clearance rates were 87.5% in the LEEP group, significantly higher than those in the laser (70.4%) and observation (60.0%) group (χ² = 11.247, P = 0.005). The histological cure rate remained highest in the LEEP group (95.2%), significantly exceeding the laser (88.9%) and the observation (66.7%) groups (χ² = 16.508, P = 0.001), with no statistically significant difference between the latter two (χ² = 3.088, P = 0.079). In the observation group, 9 (60.0%) tested negative for HPV, while 6 (40.0%) remained positive. 12-month biopsy confirmed normal tissue in 10 (66.7%), LSIL in 3 (20.0%), and persistent CIN2 in 2 (13.3%) (**Table 3**).

### Univariate analysis of persistent high-risk HPV infection in the observation group

No significant differences were observed between the two groups in general clinical data such as age, number of pregnancies, number of deliveries, age at first sexual intercourse, HPV type, HPV infection classification, smoking, and contraceptive methods (all P>0.05). However, significant differences were found in number of sexual partners and reproductive tract inflammation (all P<0.05) (**Table 4**).

**Table 4:** Comparison of general information between the two groups in the observed patient cohort.

Variable	HPV persistent infection group (n=6) (%)	HPV conversion group (n=9) (%)	t/χ2	P-value
Age (years)	22.3 ± 1.5	23.0 ± 1.1	0.987	0.341
Gravidity (times)	0.5 ± 0.8	0.1 ± 0.3	1.270	0.226
Parity (times)	0.0 ± 0.0	0.1 ± 0.3	0.806	0.435
Age at first sexual behavior	19.7 ± 1.5	19.9 ± 1.4		
HPV type				
HPV 16/18	6 (100.0)	6 (66.7)	2.500	0.114
Other HR HPVs	0 (0.0)	3 (33.3)		
HPV Infection classification				
Single infection	3 (50.0)	4 (44.4)	0.045	0.833
Multiple infection	3 (50.0)	5 (55.6)		
Smoking history				
Yes	1 (16.7)	1 (11.1)	0.096	0.756
No	5 (83.3)	8 (88.9)		
Contraceptive methods				
Condom	1 (16.7)	4 (44.4)	1.250	0.264
Oral contraceptive pills	5 (83.3)	5 (55.6)		
Number of sexual partners				
1	1 (16.7)	6 (66.7)	3.864	0.049
≥2	5 (83.3)	3 (33.3)		
Reproductive tract inflammation				
Yes	5 (16.7)	1 (11.1)	7.824	0.005
No	1 (83.3)	8 (88.9)		

### Multivariate analysis of persistent high-risk HPV infection in the observation group

Variables with a P-value < 0.05 from the univariate analyses were included in the multivariate Logistic regression model. The results of the multivariate Logistic regression analysis showed that reproductive tract inflammation (95% CI: 1.178-5.128; P = 0.039) was independent risk factors for persistent high-risk HPV infection. In contrast, multiple sexual partners showed no significant correlation with the persistence of high-risk HPV infection (95% CI: 0.010-6.233; P = 0. 395) (**Table 5**).

**Table 5:** Multivariate logistic regression analysis of persistent high-risk HPV infection in the observation group.

Independent variable	B	SE	Wald-χ2	P	95% CI
Reproductive Tract Inflammation	3.259	1.579	4.259	0.039	26.030 (1.178-5.128)
Multiple sexual partners	1.401	1.648	0.722	0.395	0.246 (0.010-6.233)

## Discussion

The reported annual incidence rates of CIN2 and CIN3 among women aged 21–24 are 3.8‰ and 4.1‰, respectively (6). Consistent with the generally asymptomatic nature of cervical precancer, the majority of patients in our cohort (86.7%, 182/210) were asymptomatic. Among the 28 symptomatic cases (13.3%), postcoital bleeding accounted for 10.0%, and abnormal vaginal discharge for 3.3%. All patients reporting discharge were co-diagnosed with vaginitis. These findings underscore that while cervical HSIL is largely asymptomatic, a subset of patients may present with postcoital bleeding or vaginitis-related discharge.

Notably, 90.0% of HSIL diagnoses in our study were established during routine gynecological examinations, whereas only 10.0% were prompted by symptoms such as postcoital bleeding. Obstetrical history further revealed that 71.4% of patients had never been pregnant, and 91.4% were nulliparous, with only 8.6% having a history of childbirth. Colposcopically, HSIL lesions exhibited a predominantly multifocal distribution, often involving two or more cervical quadrants.

In 2012, the ASCCP recommended initiating cervical cytology screening at age 21 for sexually active women, with co-testing (cytology combined HPV testing) introduced from age 30 (3). In the present study, however, 38.1% of patients under 25 with cervical HSIL showed negative cytology results. The spectrum of abnormal cytological findings included ASCUS(30.5%), LSIL(21.0%), HSIL(7.1%), and ASC-H(3.3%), with the majority of cases classified as NILM or ASCUS. Cytology alone would have identified only 7.1% of HSIL cases, thereby missing a substantial proportion of patients with underlying HSIL who presented with negative cytology—a finding consistent with previous reports (4). This underscores the limitations of ThinPrep cytology test (TCT), which is susceptible to high false-negative rates due to factors such as cellular overlap, obscuring inflammation, and inadequate sampling.

In 2015, the ASCCP endorsed primary HPV testing as the preferred screening strategy for women aged 25 and older (5). Accordingly, clinical practice currently reserves high-risk HPV testing for women ≥25 years, not recommending it for those under 25. Although HPV infection is highly prevalent (30–50%) among sexually active young women (7), most infections clear spontaneously within 8–24 months (8). In our cohort, high-risk HPV positivity reached 98.1% among cervical HSIL patients, with HPV 16 detected in 65.2%, HPV 18 in 11.0%, and HPV 16/18 co-infection in 76.15%. These results align with existing evidence that HPV is detectable in approximately 95% of HSIL specimens (9), and that HPV 16 and 18 account for about 70% of cervical cancers and 50% of precancerous lesions (10). Persistent high-risk HPV infection is a well-established driver of cervical precancer and cancer. HPV vaccination has been demonstrated to reduce the risk of high-grade cervical intraepithelial neoplasia (CIN2+) and cervical cancer. Reassuringly, a systematic review and meta-analysis by Ferrari et al. found no significant association between HPV vaccination and the development of autoimmune diseases (11). Therefore, young women can prevent HPV infection and related diseases through HPV vaccination.

In this study, high-risk HPV testing significantly improved screening sensitivity, achieving 98.1% for detecting cervical HSIL—markedly higher than cytology alone. Co-testing (HPV combined cytology) reached a sensitivity of 98.6%, also significantly superior to cytology alone. Therefore, for sexually active women under 25, incorporating HPV testing alongside cytology could substantially enhance the detection of cervical HSIL, facilitating earlier diagnosis and timely intervention.

The management of cervical HSIL in women under 25 years differs from that in older patients. Younger women with HSIL demonstrate higher rates of spontaneous regression and a lower risk of progression to invasive carcinoma compared to those aged ≥25 years (12, 13). Studies estimate the one-year progression risk to cancer among CIN3 patients aged 20–24 years to be approximately 0.5% (14). Owing to the favorable regression profile in this population, the 2019 ASCCP Risk-Based Management Consensus Guidelines endorse observation as a viable management option for patients under 25 with HSIL, provided the squamocolumnar junction is fully visible and endocervical sampling shows no evidence of CIN2+ or ungraded CIN (15). For histologically unspecified HSIL (reported as HSIL or HSIL encompassing CIN2/3), either observation or treatment is acceptable, however, intervention is recommended if CIN2 or unspecified HSIL persists beyond two years.

In the present study, all 210 cases were histologically confirmed as cervical HSIL by colposcopy-directed biopsy, including 168 cases of unspecified HSIL and 42 cases of CIN2. All patients with unspecified HSIL underwent loop electrosurgical excision procedure (LEEP), while among the CIN2 patients, 27 chose CO_2_ laser ablation and 15 opted for observation. In the observation group, the histological cure rate for CIN2 were 53.3% at 6 months and 66.7% at 12 months, with no cases progressing to CIN3 or invasive cancer. These findings are consistent with previous reports. A systematic review of CIN2 managed conservatively indicated that 50% of cases regressed, 32% persisted, and 18% progressed to CIN3, with most regressions occurring within 12 months and progression risk increasing over time. Regression rates were higher in women under 30, reaching approximately 60.0% (16). Lee et al. (17) reported a 55.2% regression rate within two years among patients under 24 with conservatively managed cervical HSIL, with a persistence rate of 44.8%. Similarly, Loopik et al. (18) observed that among CIN2 patients under 25 followed for a mean of 15.1 months, 71.1% regressed, 12.3% persisted, and 16.6% progressed to CIN3.

In our cohort, the persistent high-risk HPV infection rate in the observation group was 40.0% at the 1-year follow-up. We also identified that reproductive tract inflammation as a significant risk factor for HPV persistence, corroborating findings by Juan Wang (19). who reported reproductive tract inflammation as an independent risk factors for persistent high-risk HPV infection in patients with CIN. Inflammation disrupts the cervical microenvironment and compromises local immune function (20), impairing viral clearance and facilitating persistent HPV infection. Meanwhile, mucosal changes induced by inflammation—such as increased permeability and impaired epithelial barrier function—can enhance HPV attachment and invasion (21). In addition, the inflammatory mediators and cytokines released during the inflammatory process may also promote the persistent existence of high-risk HPV infection (19). Although previous studies have linked smoking and multiple sexual partners to HR-HPV persistence (22), we did not observe a significant association in our cohort. This discrepancy may be attributable to the limited sample size of the observation group (n=15). Future studies with larger cohorts are warranted to clarify the role of these factors in persistent HPV infection among young women with HSIL.

Among patients treated with LEEP, postoperative histopathology confirmed HSIL in 88.7% of cases, while 8.3% were downgraded to LSIL and 3.0% showed normal cervical tissue. None of the cases progressed to cervical cancer. The observed pathological downgrading following LEEP may be attributed to two potential factors: first, the original HSIL lesion may have been small and possibly entirely excised during the initial diagnostic biopsy; second, the HSIL focus might have been minimal and superficial, rendering it susceptible to disruption by the thermal artifact induced by the electrosurgical loop during excision. The negative margin rate was 91.1%, with positive margins observed in 8.9% of cases, and all instances of positive margins were located on the ectocervix, which indicates that LEEP achieves a high rate of complete excision and a low positive margin rate in young patients with cervical HSIL. For patients with positive margins, close follow-up should be conducted.

At the 6-month follow-up, normal cytology rates were 87.5% in the LEEP group, compared to 70.4% in the laser group and 53.3% in the observation group. The HPV clearance rate was 64.9% in the LEEP group and 55.6% in the laser group, both significantly higher than the 13.3% rate in the observation group; however, no statistically significant difference in HPV clearance was observed between the LEEP and laser groups. The histological cure rate was highest in the LEEP group (94.0%), significantly exceeding that in the laser (77.8%) and observation (53.3%) groups, while no significant difference was observed between the laser and observation groups.

By the 12-month follow-up, the LEEP group continued to demonstrate superior outcomes, with the highest rates of normal cytology (90.5%), HPV clearance (87.5%), and histologic cure (95.2%), consistently outperforming both the laser and observation groups.

This study demonstrates that LEEP achieves the highest rates of normal cytology, HPV clearance and histological cure for cervical HSIL in patients under 25, establishing it as the preferred treatment for young patients who have completed childbearing, given its superior efficacy in preventing cervical cancer progression. However, LEEP is associated with an increased risk of adverse obstetric outcomes, including mid-trimester pregnancy loss, preterm premature rupture of membranes, and preterm delivery (23–25). Cold knife (CK) conization and carbon dioxide (CO2) laser conization are two established techniques for excising pre-invasive cervical lesions. Ferrari F et al. (26) reported that CO2 laser conization achieved a lower rate of positive endocervical or deep margins compared to CK conization, suggesting it is a viable alternative for managing HSIL. It should be noted, however, that the laser modality employed in the present study was CO_2_ laser ablation rather than laser conization, thereby precluding a direct pathological comparison between laser conization and LEEP. Future prospective studies are warranted to compare the therapeutic outcomes of CO_2_ laser conization, LEEP, and cold knife conization in treating HSIL.

For nulliparous patients under 25, a more conservative management approach is recommended. When concerns regarding potential fertility impacts outweigh the risk of cervical cancer progression, active observation may be considered-provided the squamocolumnar junction is fully visible and endocervical sampling shows no CIN2+ or ungraded CIN. Patients should be thoroughly counseled regarding the risks of persistent HPV infection during observational management. Close monitoring through HPV testing, cervical cytology (TCT), and colposcopy is essential throughout this period. Timely treatment of reproductive tract inflammation is also crucial to support HPV clearance. Should CIN2 or unspecified HSIL persist for more than two years, active treatment is recommended.

This study has several limitations. The sample sizes for the laser therapy and observation groups were relatively small, which may affect the generalizability of the findings. In addition, the clinical follow-up was limited to 12 months post-treatment, restricting our ability to analyze long-term outcomes. The truncated follow-up period was largely attributable to patients transitioning to local healthcare facilities after initial normal results, as well as loss to follow-up due to financial constraints. Consequently, the absence of 24-month postoperative data precluded its inclusion in the present analysis. Future multicenter, large-sample, prospective controlled studies are needed to comprehensively evaluate the clinical outcomes of observation, laser therapy, and LEEP for cervical HSIL in patients under 25, thereby providing a robust evidence base for optimizing treatment in this population.

In conclusion, the majority of cervical HSIL patients under 25 are asymptomatic at presentation. While cytology alone demonstrates limited sensitivity for reliable detection, HPV-based screening significantly improves sensitivity. In terms of treatment, LEEP achieves the highest histological cure rates, though its potential impact on future pregnancy outcomes warrants careful consideration in nulliparous women. Additionally, reproductive tract inflammation represents an independent risk factor for persistent high-risk HPV infection among HSIL patients under observational management.

## Data Availability

The raw data supporting the conclusions of this article will be made available by the authors, without undue reservation.
